# Developing a *de novo* targeted knock-in method based on *in utero* electroporation into the mammalian brain

**DOI:** 10.1242/dev.136325

**Published:** 2016-09-01

**Authors:** Yuji Tsunekawa, Raymond Kunikane Terhune, Ikumi Fujita, Atsunori Shitamukai, Taeko Suetsugu, Fumio Matsuzaki

**Affiliations:** Laboratory for Cell Asymmetry, RIKEN Center for Developmental Biology, 2-2-3 Minatojima-Minamimachi, Chuo-ku, Kobe 650-0047, Japan

**Keywords:** *CRISPR*, *CAS9*, *In utero* electroporation, Gene knock-in, Lineage tracing, Ferret, Mouse

## Abstract

Genome-editing technology has revolutionized the field of biology. Here, we report a novel *de novo* gene-targeting method mediated by *in utero* electroporation into the developing mammalian brain. Electroporation of donor DNA with the CRISPR/Cas9 system vectors successfully leads to knock-in of the donor sequence, such as *EGFP*, to the target site via the homology-directed repair mechanism. We developed a targeting vector system optimized to prevent anomalous leaky expression of the donor gene from the plasmid, which otherwise often occurs depending on the donor sequence. The knock-in efficiency of the electroporated progenitors reached up to 40% in the early stage and 20% in the late stage of the developing mouse brain. Furthermore, we inserted different fluorescent markers into the target gene in each homologous chromosome, successfully distinguishing homozygous knock-in cells by color. We also applied this *de novo* gene targeting to the ferret model for the study of complex mammalian brains. Our results demonstrate that this technique is widely applicable for monitoring gene expression, visualizing protein localization, lineage analysis and gene knockout, all at the single-cell level, in developmental tissues.

## Abstract (precis)

**Summary:** A novel targeted knock-in technique based on CRISPR/Cas9 and *in utero* electroporation enables detection of homozygous knock-in based on dual fluorescent reporters in both mouse and ferret.

## INTRODUCTION

*In utero* electroporation is a laboratory technique widely used to introduce transgenes into tissues in developmental biology studies, especially in brain development (Fukuchi-Shimogori and Grove, 2001; Saito and Nakatsuji, 2001; Tabata and Nakajima, 2001). This technique has provided various useful toolkits that enable the modification of gene function in brain tissue by overexpression, misexpression and knockdown of genes (Mellitzer et al., 2002; Nakamura and Funahashi, 2013; Ochiai et al., 1998), as well as visualization of the progeny of progenitor cells both in fixed samples and in live imaging (Pilz et al., 2013; Shitamukai et al., 2011). However, the *in vivo* manipulation of a particular gene in the genome is difficult using the electroporation technique. Making conventional knock-in (KI) or knockout (KO) animals has been the most reliable approach for this purpose.

Recently, novel genome-editing technologies have been developed to accelerate the generation of genetically modified animals. These technologies rely on insertion or deletion at genomic target sites via non-homologous end joining (NHEJ) following the mRNA/DNA/protein injection of site-specific nucleases, such as zinc finger nucleases (ZFNs), transcription activator-like effector nucleases (TALENs) and clustered regularly interspaced short palindromic repeats (CRISPR)-associated protein 9 (Cas9), into one-cell-stage embryos (Carbery et al., 2010; Geurts et al., 2009; Hai et al., 2014; Kou et al., 2015; Ni et al., 2014; Niu et al., 2014; Sung et al., 2013; Tesson et al., 2011). Furthermore, KI mice have also been made via homology-directed repair (HDR) by injecting site-specific nucleases with donor DNA into one-cell-stage embryos (Aida et al., 2015). If these genome-editing technologies can be combined with the electroporation method, *in vivo* manipulation such as KI and KO of a particular gene can be achieved. Indeed, it was recently reported that *in vivo* gene KO occurs efficiently when mediated by CRISPR/Cas9 delivered via *in utero* electroporation (Chen et al., 2015; Kalebic et al., 2016; Straub et al., 2014). Likewise, targeted gene KI via *in utero* electroporation will provide various advantages for developmental studies, including precise tracing of cell lineage, visualization of the localization of a knocked-in gene product, and also identification of cells homozygous for gene knockout, all at the single-cell level, not only in model animals but also in non-model animals that are not suitable for conventional gene KI strategy.

Here, we report a new technique that allows targeting of gene KI to neural progenitors by delivering the CRISPR/Cas9 system into the developing mammalian cortex by *in utero* electroporation.

## RESULTS AND DISCUSSION

### Homology-directed repair-mediated *de novo* gene KI in mouse neural progenitors

**Fig. 1.**
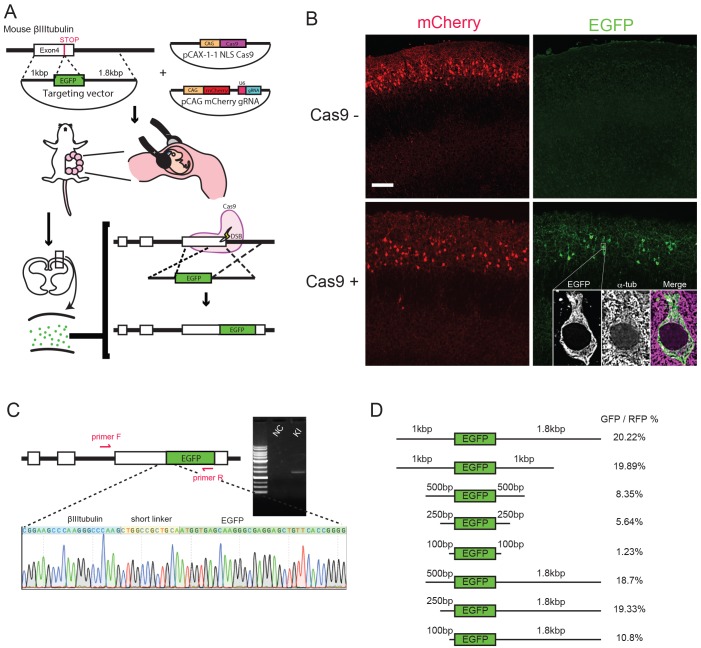
***De novo* gene KI mediated by *in utero* electroporation.** (A) Schematic of *de novo* targeted KI of developing mouse brain cells by *in utero* electroporation. See the text for details. (B) P10 dorsal cortex electroporated with the three types of vectors at E15.5. Many mCherry-positive (electroporated) neurons were EGFP positive when co-electroporated with Cas9 (bottom right), but not without the electroporation of the Cas9 vector (top right). EGFP distribution showed a good correlation with microtubule meshworks visualized by α-tubulin staining (bottom right, inset). The image was processed with deconvolution. (C) Genomic DNA was extracted from the electroporated brains. The PCR fragment containing the junction of the genomic DNA with the donor DNA (primer F/R) was amplified only from the genomic DNA of the KI brain. Seven independent clones of the amplicons showed the expected sequence (7/7). NC, negative control. (D) KI efficiency was calculated as the proportion of EGFP-positive cells out of mCherry-positive cells; the individual KI efficiencies were 20.22±5.9% (mean±s.d.) (*n*=4 embryos; 17.97%, 27.11%, 13.42% and 22.37%) for 1 kb/1.8 kb; 19.89±5.7% (*n*=5 embryos; 23.43%, 19.07%, 16.44%, 13.09% and 27.44%) for 1 kb/1 kb; 8.35±1.5% (*n*=6 embryos; 6.35%, 10.90%, 8.55%, 8.14%, 8.04% and 8.10%) for 500 bp/500 bp; 5.64±2.7% (*n*=6 embryos; 1.46%, 8.65%, 8.29%, 4.24%, 4.78% and 6.40%) for 250 bp/250 bp; 1.23±0.5% (*n*=3 embryos; 1.83%, 0.75% and 1.11%) for 100 bp/100 bp; 18.7±2% (*n*=6 embryos; 19.75%, 19.54%, 16.03%, 18.98%, 21.24% and 16.63%) for 500 bp/1.8 kb; 19.33±3.8% (*n*=6 embryos; 15.97%, 23.68%, 13.75%, 22.28%, 20.20% and 20.08%) for 250 bp/1.8 kb; and 10.8±2.2% (*n*=5 embryos; 9.57%, 10.35%, 11.66%, 8.29% and 14.15%) for 100 bp/1.8 kb. Scale bar: 100 μm.

To develop the *de novo* knock-in method based on *in utero* electroporation, we first examined whether HDR can mediate gene KI in mouse neural progenitors. We designed guide RNA (gRNA) against the fourth exon of βIII-tubulin (*Tubb3*). The targeting vector was constructed from the *EGFP* gene flanked by short homology arms (1 kb and 1.8 kb) so that the *EGFP* gene is inserted into mouse *Tubb3* to produce EGFP fused in-frame with the C terminus of the mouse Tubb3 protein (Fig. 1A). The inserted donor sequence has no gRNA target sequence, and hence the targeted allele is no longer affected by Cas9. The targeting vector, pCAX-Cas9 expression vector and pCAG-mCherry-gRNA vector were co-electroporated into embryonic day (E) 15.5 mouse embryos. Omission of the Cas9 expression vector was used as a control. When the pups' brains were fixed and observed at postnatal day (P) 10, EGFP-expressing cells were found only in the brains electroporated with all three types of expression vectors, indicating that Cas9 mediates double-strand break (DSB)-induced HDR with the targeted plasmid sequence (Fig. 1B, inset). PCR amplification of the junction of *Tubb3* and the donor *EGFP* was detected only for genomic DNA from the Cas9-electroporated brains and not from the control brains (Fig. 1C). The sequencing of seven independent clones of the DNA fragment revealed that HDR-mediated gene KI occurred correctly in mouse neural progenitors (Fig. 1C). We next examined the efficiency of *de novo* gene KI via *in utero* electroporation by testing targeting vectors for the *Tubb3* fusion gene carrying various lengths of the homology arms from 100 bp to 1.8 kb (Fig. 1D). The KI efficiency, measured as the percentage of EGFP-positive cells out of mCherry-positive cells, essentially remained unchanged when both arms were longer than 1 kb but gradually decreased when both arms were 500 bp or shorter, dropping to 1% for the arm pair of 100 bp-100 bp (Fig. 1D). Interestingly, the targeting vectors with a long 5′ arm and a short 3′ arm showed KI efficiencies comparable to the control (Fig. 1D). This property is useful because (1) PCR amplification to confirm precise KI is easier with a short arm, and (2) donor plasmid leakage occurs less frequently for a shorter 5′ arm (see below; Table S2).

### Optimization of targeting vectors to minimize leakage

**Fig. 2.**
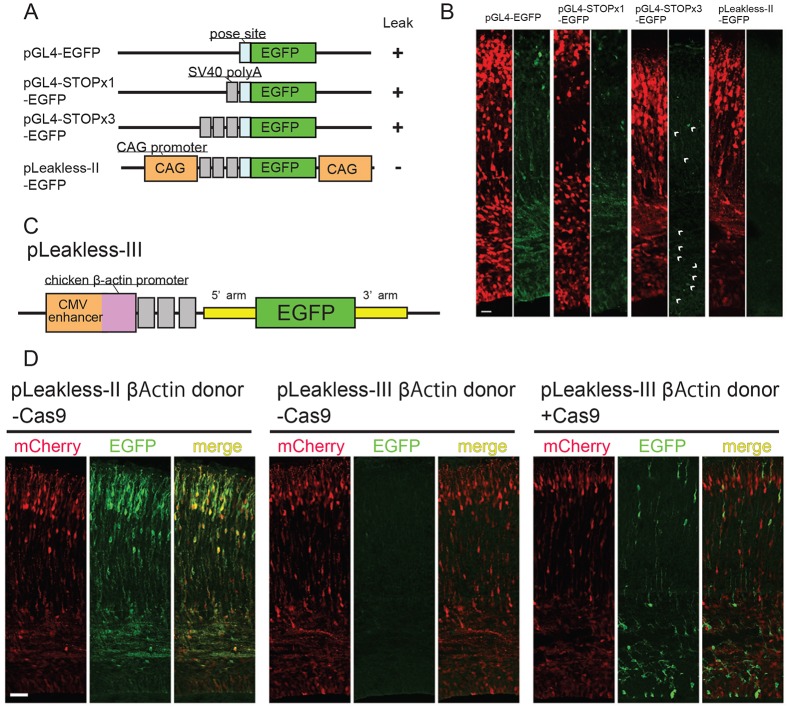
**Leakless backbone vector construction.** (A) Schematic of backbone vectors. (B) Vectors listed in Fig. 2A were electroporated at E13.5 with the mCherry expression vectors and fixed at E17.5. All backbone vectors except pLeakless-II exhibited EGFP leakage. Arrowheads indicate EGFP leakage signals. (C) Schematic of pLeakless-III. (D) The mouse *Actb* donor in pLeakless-II/III, mCherry expression vector and gRNA vector were electroporated with/without Cas9 expression vector at E13.5, and fixed at E17.5. The pLeakless-II EGFP donor showed a leaky expression without Cas9 (left). The pLeakless-III donor gave no leaky EGFP without Cas9 (middle), but clear EGFP signals with Cas9 (right). Some EGFP-positive cells showed faint mCherry staining owing to the dilution of the vector after multiple divisions. Scale bars: 20 μm (B); 40 μm (D).

The donor gene should be completely suppressed unless knocked-in to the genome, but we often observed a leaky expression of the donor gene even in the absence of the Cas9 vector (Table S2). This leakage from the plasmid might be due to either anomalous transcription activity of the backbone vector or promoter activity in the 5′-arm in front of the knock-in sequence. To prevent this leakage, the backbone of the targeting vector was optimized using the pGL4.23 plasmid designed for the luciferase assay of promoter activities with no anomalous transcription (Fig. 2A). This vector worked well as the *Tubb3* targeting vector (Fig. 1A). However, pGL4.23 carrying only the *EGFP* gene showed leaky expression in embryos even without Cas9 and gRNA (Fig. 2B). Inserting several STOP cassettes (SV40 polyA signal) upstream of the *EGFP* gene (Fig. 2A) failed to eliminate leakage completely (Fig. 2B). Additional insertions of *CAG* promoters upstream of the STOP cassettes and downstream of the *EGFP* gene successfully suppressed *EGFP* leakiness, perhaps by sequestering transcriptional machineries (Fig. 2A,B, pLeakless-II vector); the *CAG* promoter is a strong promoter combining the human cytomegalovirus (CMV) early enhancer and chicken β-actin (*ACTB*) promoter followed by the first exon and intron (Miyazaki et al., 1989; Niwa et al., 1991). Because this vector, including some 5′ and 3′ homology arms, still caused leakage (Table S2), we finally deleted the splicing donor of the chicken *ACTB* first intron and the following sequence from the *CAG* promoter to exclude the splicing-out of the STOP cassettes between this splicing donor and the possible splicing acceptor within the 5′ genomic arm. The resulting pLeakless-III vector (Fig. 2C) quenched donor leakage well with 5′-homology arms that caused leakage in the pLeakless-II vector. We show the case of the β-actin (*Actb*) gene as an example (Fig. 2D; Table S2). In the following experiments, we used pLeakless-III as the targeting vector when the pLeakless-II vector showed donor leakage.

### *De novo* KI into various target genes

To examine further whether *de novo* targeting could be applicable to various types of genes, we targeted the *Eomes* (*Tbr2*) and *Pax6* transcription factor genes, expressed in transient intermediate progenitors and self-renewing progenitors, respectively. The targeting vectors for the *Tbr2* and *Pax6* were constructed to fuse the C terminus of the Tbr2 and Pax6 proteins with EGFP. EGFP was detected exclusively in the Tbr2-positive and Pax6-positive nuclei at the subventricular zone and ventricular zone only when co-electroporated with Cas9 (Fig. S1A,B), and a neuronal migration defect was observed for *Tbr2*-*EGFP* KI. Because HDR-mediated gene KI occurs in the S phase of proliferating cells and not in post-mitotic cells (Karanam et al., 2012), these data suggest that HDR-mediated gene KI occurs efficiently in mouse neural progenitors.

### Homozygous *de novo* KI via *in utero* electroporation in neural progenitors

**Fig. 3.**
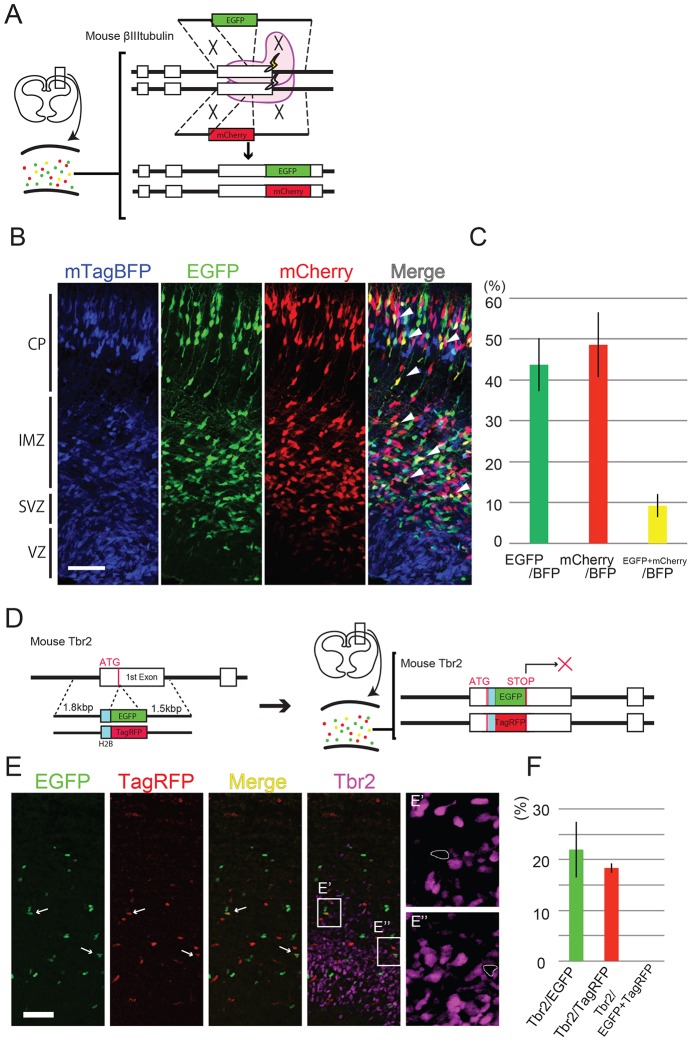
**Homozygous *de novo* KI by *in utero* electroporation.** (A) Schematic of homozygous KI of dual fluorescent marker genes into the *Tubb3* gene. Some cells show the yellow fluorescent color reflecting the insertion of both EGFP and mCherry in homologous chromosomes. (B) EGFP/mCherry double-positive cells were observed (arrowheads). CP, cortical plate; IMZ, intermediate zone; SVZ, subventricular zone; VZ, ventricular zone. (C) Percentage of EGFP-positive, mCherry-positive and EGFP/mCherry double-positive cells out of total BFP-positive cells were 43.74±6.47%, 48.62±7.89% and 9.21±2.85%, respectively, the outside of the VZ (*n*=6 embryos). (D) Schematic of homozygous KO of two *Tbr2* alleles by *de novo* KI. (E-E″) No Tbr2 expression was observed in EGFP/TagRFP double-positive cells (arrows; E′,E″). Double-positive cells are circled in E′ and E″. (F) Out of total EGFP-positive cells, 21.99±5.5% were Tbr2 positive (*n*=4 embryos; 23.35%, 24.76%, 25.95% and 13.9%) and out of total TagRFP-positive cells 18.36±0.9% were Tbr2 positive (*n*=4 embryos; 18.64%, 17.37%, 17.94% and 19.5%). Because EGFP (TagRFP) is fused with H2B, and thereby localized to chromosomes, the progeny of the knocked-in cells retains EGFP (TagRFP) after the termination of Tbr2 expression. By contrast, Tbr2-positive cells were never found in EGFP/TagRFP-double positive cells. Error bars indicate s.d. Scale bars: 50 μm.

The high KI efficiencies of our *de novo* KI strategy for brain cortical cells predict that some targeted cells are homozygous for KI. To identify cells homozygous for knocked-in alleles, we used two targeting vectors for Tubb3 C-terminus fusion simultaneously, each with EGFP or mCherry (both following a self-cleaving peptide 2A) (Fig. 3A). We simultaneously electroporated E12.5 embryos with these two targeting vectors along with Cas9 and mTagBFP-gRNA expression vectors (to mark electroporated cells with BFP). Two (Fig. S2) or 3 days later (Fig. 3B,C), we found that approximately 10% of the electroporated cells (BFP positive) expressed both EGFP and mCherry (hereafter called yellow cells), indicating that *Tubb3* was homozygously targeted by EGFP and mCherry in those cells. Our mathematical model explains that this double-color KI efficiency is reasonably derived from the efficiency of each single color (supplementary Materials and Methods; Fig. S4). The KI efficiencies were higher with electroporation at E12.5 than that at E15.5 (Fig. 1D; Fig. 3C), suggesting that the neural progenitors at an early developmental stage are more susceptible to KI, perhaps owing to their higher proliferative activity (and, therefore, higher HDR efficiency) at the early stages.

We next attempted to make cells homozygous for the *Tbr2* gene KO as a model target locus. We designed the targeting vectors and gRNA vector so that the fusion of *Histone H2B* (*H2B*)-*EGFP* and *H2B*-*TagRFP* directly follow the *Tbr2* start codon (see Fig. 3D legend for details). Cells carrying the correct insertion of both *H2B-EGFP* and *H2B-TagRFP* at the two *Tbr2* alleles should not express *Tbr2* but *H2B-EGFP* and *H2B-TagRFP* under the endogenous *Tbr2* promoter (Fig. 3D). When we examined fluorescent protein expression 3 days after E12.5 electroporation of the vector set, we found that approximately 20% of EGFP single-positive cells and TagRFP single-positive cells were Tbr2 positive, indicating that at least one fifth of those cells were heterozygous for *Tbr2* KO (details are described in Fig. 3E,F legend). In addition, ‘yellow cells’ (EGFP/TagRFP double positive) were generated (4.37±0.85% of EGFP-positive cells and 7.58±1.29% of TagRFP-positive cells). As expected, these yellow cells were never Tbr2 positive (Fig. 3E, arrows, E′,E″,F), compared with EGFP (or TagRFP) single-positive cells. These results validate our strategy.

### *De novo* gene KI application in the developing ferret brain

**Fig. 4.**
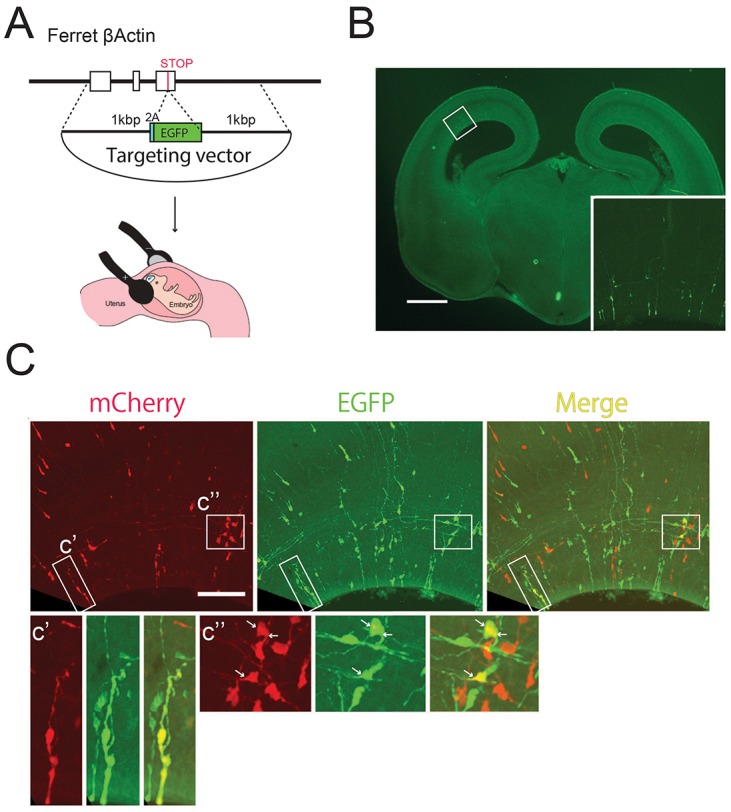
***De novo* KI in the embryonic ferret brain.** (A) Schematic of *de novo* targeted KI to the developing ferret brain by *in utero* electroporation. The targeting vector was electroporated into ferret embryos with the ubiquitous expression vector for Cas9 and gRNA. (B) The dorsal cortices of E32 ferret embryos electroporated with the described vectors *in utero* and fixed at E36. EGFP signals were observed in the offspring of electroporated neural progenitors (inset). Vibratome sections: 200 μm. (C) Embryos electroporated with EGFP and mCherry targeting vectors against *ACTB* simultaneously, together with ubiquitous expression vectors for Cas9 and gRNA at E32 and fixed at E36. EGFP/mCherry-double positive cells formed sparsely distributed cell clusters (five cell clusters out of ten slices), indicating they were clonal (C′,C″ arrows). Scale bars: 1 mm (B); 100 μm (C).

Finally, we examined whether our *de novo* gene KI method is applicable to other mammalian models. The ferret (*Mustela putorius furo*) has been used as a model for a more complex brain than rodent brains, with gyri (Borrell, 2010; Chenn and McConnell, 1995; de Juan Romero et al., 2015; Fietz et al., 2010; Nonaka-Kinoshita et al., 2013; Poluch and Juliano, 2015; Reillo and Borrell, 2012; Ware et al., 1999). The ubiquitously expressed ferret β-actin gene *ACTB* was targeted with a 2A-EGFP targeting vector with short homology arms (1 kb/1 kb) to generate the *ACTB-2A-EGFP* fusion gene, thereby producing EGFP in all knocked-in cells (Fig. 4A). EGFP-expressing cells were radially aligned 4 days after electroporation of E32 ferret embryos with this targeting vector, along with Cas9 and gRNA expression vectors. These individual cell clusters are most likely the offspring of single targeted neural progenitors (Fig. 4B, inset). The genomic sequencing of fluorescence-activated cell sorting (FACS)-sorted EGFP-positive cells showed that HDR-mediated gene KI occurred properly (Fig. S3). We also confirmed that homozygous *de novo* KI via *in utero* electroporation worked in the ferret developing brain by the electroporation of E34 embryos with Cas9 and gRNA expression vectors along with *2A-EGFP* and *2A-mCherry* targeting vectors against *ACTB*. Double-color targeted cell clusters were sparse enough (five clusters out of ten slices of hemispheres) to label the clone from a single cell (Fig. 4C,C′,C″, arrows). Thus, our *de novo* gene KI method is applicable to the ferret model.

### Future applications of *de novo* targeted knock-in method

We showed that our new practical method for *de novo* targeted KI could be applicable for many purposes and occasions: (1) detection of endogenous subcellular protein localization, (2) visualization of homozygous acute gene knockout cells, (3) quick and directed lineage tracing, and (4) applications for non-rodent model animals.

However, our current protocol is not practical for KI of the *Cre* gene. Leakage of a very small amount of Cre protein is inevitable from the electroporated targeting vector carrying the *Cre* gene and will function before integrating into the target genome. Although more improvements could be made for wider applications of this technique, the current advantages of recognizing homozygous KI cells by double-color labeling makes our *de novo* KI method unique and useful, especially for the visualization of homozygous KO cells and lineage analysis. The use of more fluorescent protein genes as donors will be a fascinating expansion to this *de novo* KI method based on the CRISPR/Cas9 system.

## MATERIALS AND METHODS

### Animals

ICR mice were used for all experiments. Embryonic stages were calculated using noon on the day of the vaginal plug as E0.5. Some mice were purchased from Japan SLC (Fukuoka, Japan). Ferrets were purchased from Marshall Bioresources (New York, USA). All experiments were performed in compliance with the guidelines for animal experiments at the RIKEN Center for Developmental Biology.

### Plasmid construction

The guide RNAs (gRNAs) against all target genes (each 20 bp target sequence) were designed using the Zhang lab website (http://crispr.mit.edu/) (Cong et al., 2013), and DNA oligonucleotides were obtained from Hokkaido System Science. The gRNA fragment was amplified by PCR through self-amplification of the designed primer set and cloned into *Afl*II-cut gRNA vectors (mCherry-gRNA and mTagBFP-gRNA) modified from the original Church lab vector (DiCarlo et al., 2013). All primers used to construct gRNAs and targeting vectors are listed in Table S1.

For targeting vector construction, the EGFP fragment of pCAX-EGFP (Tsunekawa et al., 2012) was PCR-amplified, flanked by homology arms (amplified from the mouse or ferret genomes), and inserted using In-Fusion (Takara, Japan) into *Bam*HI/*Hin*dIII-cut pGL4.23 (Promega), *Xho*I/*Kpn*I-cut pLeakless-II or *Eco*RI/*Kpn*I-cut pLeakless-III vector. For pGL4-EGFP, the fragment of pCAX-EGFP was amplified using primer pair A and inserted into *Bam*HI/*Hin*dIII-cut pGL4.23 by In-Fusion. For pGL4-STOPx1-EGFP, the fragment of pCAG-flox-STOP-EGFP was amplified using primer pair B and inserted into *Nhe*I/*Xho*I-cut pGL4-EGFP by In-Fusion. For pGL4-STOPx3-EGFP, the fragment of pCAG-Roxed-Cre (addgene #51273) was amplified using primer pair C and inserted into *Xho*I/*Kpn*I-cut pGL4-EGFP by In-Fusion. For pLeakless, the *Hin*cII/*Xba*I-cut fragment of pCAX was inserted into *Eco*RV/*Xba*I-cut pGL4.23 by ligation. The vector pLeakless-II was constructed by sequential insertion of the CAG (Niwa et al., 1991)-promoter-3x polyA fragment (amplified by PCR from pCAG-Roxed-Cre) and the CAG-promoter-polyA fragment from pCAX into pGL4.23. For pLeakless-III, the fragment of pCAX (amplified using primer pair D) and the fragment of pCAG-Roxed-Cre (amplified using primer pair E) were inserted into *Sal*I/*Eco*RI-cut pCAX by In-Fusion. Primer pairs are listed in Table S1.

### *In utero* electroporation

Mouse *in utero* electroporation was performed as previously described (Konno et al., 2008). Detailed conditions are presented in the supplementary Materials and Methods. For ferret *in utero* electroporation (Kawasaki et al., 2013, Matsui et al., 2013), pregnant ferrets were anesthetized with isoflurane. Embryonic brain hemispheres were injected with 4 µl of the DNA solution as described above and 0.005% Fast Green FCF (Wako, Japan). Embryos were placed between the paddles of the electrodes (CUY21 electroporator, NEPA GENE, Japan), then subjected to 5×100 ms/55 V electric pulses.

### Immunohistochemistry

Immunohistochemistry was carried out on 12-μm-thick mouse brain sections or 200-μm-thick ferret brain slices. Detailed methods are described in the supplementary Materials and Methods.

### Tissue dissociation and fluorescence-activated cell sorting (FACS)

The ferret embryos were *in utero* electroporated at E32 with *Actb* targeting vectors and Cas9 and gRNA expression vectors, then brains were dissected at E36. Electroporated brains were dissected in the saline and trypsinized by 0.5% trypsin-EDTA (Gibco) for 40 min at 37°C. Brains were dissociated by pipetting and EGFP-positive cells were sorted using a SH800 cell sorter (Sony, Tokyo, Japan).

### Genomic DNA sequencing of KI cells

The genomic DNA was isolated from electroporated cells using a DNeasy Blood & Tissue Kit (Qiagen). The junction of the target gene and the reporter gene was amplified by PCR and amplicons were subcloned into TOPO cloning vector using the Zero Blunt TOPO Kit (Invitrogen). Clones of amplicons were isolated from each *Escherichia coli* colony using a Wizard Miniprep Kit (Promega) and sequenced using M13F and M13R primers. 

### Note added in proof

During the revision process of the manuscript, a paper describing a similar method was published (Mikuni et al., 2016).
